# *Lactobacillus fermentum* 166, Derived from Yak Yogurt from Tibetan Areas of Sichuan, Improves High-Fat-Diet-Induced Hyperlipidemia by Modulating Gut Microbiota and Liver- and Gut-Related Pathways

**DOI:** 10.3390/foods14050867

**Published:** 2025-03-03

**Authors:** Shiqi Zhang, Limei Xu, Chenglin Zhu, Jing Li, Yu Fu, Weiming Shuang, Lianhong Chen

**Affiliations:** College of Pharmacy and Food, Southwest Minzu University, Chengdu 610041, China; 220905212004@stu.swun.edu.cn (S.Z.); limei.xu2025@outlook.com (L.X.); chenglin.zhu@swun.edu.cn (C.Z.); 230905212005@stu.swun.edu.cn (J.L.); yufu@swun.edu.cn (Y.F.); 240905212003@stu.swun.edu.cn (W.S.)

**Keywords:** *Lactobacillus fermentum* 166, yak yogurt, gut microbiota, lipid accumulation, mRNA expression

## Abstract

The consumption of an unbalanced diet, such as a high-fat diet, is strongly associated with hyperlipidemia and significantly contributes to the development of cardiovascular and cerebrovascular diseases, which are the leading causes of death worldwide. Globally, about 17.9 million people die of cardiovascular disease each year (WHO 2023). Probiotics have emerged as a promising intervention to alleviate hyperlipidemia. Therefore, this study investigates the effects of *Lactobacillus fermentum* 166 (LF-166), isolated from yak yogurt in the Sichuan Tibetan area, on lipid metabolism in the liver and gut microbiota of high-fat-diet-induced hyperlipidemic mice. The results revealed that the *Lactobacillus fermentum* 166 (LF-166) treatment reduced the body weight and decreased the blood and liver lipid levels in these mice. Based on the histopathological findings, LF-166 could alleviate liver steatosis and colon injury. Additionally, 16S rRNA sequencing of the mice’s colonic contents showed that LF-166 reduced the *Firmicutes*/*Bacteroidetes* (F/B) value and enhanced the richness and diversity of the gut microbiota. LF-166 regulated hepatic lipid metabolism through the up-regulation of the genes *Lxr*, *Ampkα*, *Fxr*, *Hsl*, and *Atgl* and the down-regulation of *C/ebpα* and *Pparγ* in the liver; it also regulated intestinal lipid metabolism by up-regulating *Abcg5* and *Abcg8* in the ileum and down-regulating the expression of the genes *Npc1l1*, *Asbt*, and *Ibabp*. Thus, LF-166 may inhibit hyperlipidemia progression by modulating the expression of key genes involved in hepatic lipid metabolism, influencing the intestinal microbiota through the liver–gut axis, and regulating systemic lipid metabolism.

## 1. Introduction

Hyperlipidemia is a highly prevalent metabolic disease. It is one of the most common human pathological conditions, and its pathogenesis is associated with lipid metabolism disorders and increased plasma triglycerides or cholesterol. Epidemiological findings indicate that hyperlipidemia is one of the major causal factors contributing to cardiovascular diseases (CVDs) [1]. Based on incomplete statistics, CVDs account for approximately 33% of deaths worldwide and were responsible for approximately 40% of deaths in China from 2009 to 2019. The World Health Organization (WHO) predicts that CVDs will remain a significant cause of human death by 2030. Epidemiological findings reveal that people with hyperlipidemia have about a three times higher risk of developing CVDs than those without lipidemia [2]. Furthermore, a 1% increase in the serum cholesterol concentration causes a 2% to 3% increase in the CVD incidence. If patients are hyperlipidemic, they could develop fatty liver and atherosclerosis over time, then cardiovascular disease, cirrhosis, and coronary heart disease [3]. Therefore, it is necessary and urgent to take control measures for hyperlipidemia [4].

The intestinal microbiota and the host exhibit a highly complex symbiotic relationship. The host provides a nutrient-rich site for the bacteria to proliferate. The bacteria, in turn, provide metabolic, protective, and structural functions that are not encoded by the host genome. Therefore, the host health status is affected by the beneficial or harmful effects of intestinal microbiota associated with different physiological states of the organism. For example, *Lactobacillus paracasei* OWS153 and *Lactobacillus fermentum* OWS23 can prevent invasion by pathogenic bacteria, such as *Listeria monocytogenes* and *Staphylococcus aureus* [5]. If the host organism is in a pathological state, influenced by various pathogenic bacteria, the intestinal flora becomes out of balance, leading to more severe disease. In addition, there is mutual regulation between the gut microbiota in the GI tract and the host immune system. For example, high-fat diets lead to dysbiosis of the gut microbiota, which causes altered metabolite production and disruption of the gut barrier integrity by modulating immune cells and intestinal epithelial cells [6]. Research continues to establish that the host’s environment, dietary habits, and genetic factors can impact the intestinal microbiota, imparting diversity and specificity. Certain research findings have demonstrated that remodeling the intestinal microbiota ameliorates hyperlipidemia [7], with *Lactobacillus brevis* FZU0713 emerging as a potential probiotic strain for modulating the gut microbial composition for hyperlipidemia prevention [8]. Thus, the microbiota is likely to be an important target for the prevention and treatment of metabolic diseases.

The yak is an ancient breed of cattle found in the western Tibetan plateau areas of China, such as the Tibetan areas in Sichuan Province [9]. Furthermore, yak milk and its dairy derivatives play a pivotal role in the diet of Tibetan herders, serving as essential sources of nutritional sustenance. Compared to conventional bovine milk, yak milk exhibits comparatively lower yields yet demonstrates superior nutritional parameters, such as protein concentration, lipid content, and total solids. Furthermore, it contains a more comprehensive array of bioactive compounds and harbors distinct microbial communities [10]. The lactobacillus functions that have been identified include, for instance, *Lactobacillus fermentum* CQPC05’s anti-obesity effect on obese mice [11] and *Lactobacillus plantarum* YS4’s ability to alleviate constipation induced by activated carbon in mice [12].

LF-166 has been proven to cause a definite cholesterol-lowering effect and exhibits a good safety profile and gastrointestinal tolerance in vitro. Our previous results demonstrated that LF-166 reduces body weight, lowers blood lipids, and reduces liver fat accumulation in high-fat-diet-fed mice. Based on previous research, we used a hyperlipidemic mice model fed with a high-fat diet and measured serum and tissue indices, e.g., cholesterol and triglyceride levels. However, the lipid-lowering mechanism of fermented Lactobacillus in yak yogurt has not been reported. Therefore, we assessed the efficacy of LF-166 in reducing lipid accumulation and its beneficial effects on the gut microbiota in hyperlipidemic mice, while also conducting a preliminary investigation into its underlying mechanism of action. This provided a theoretical foundation for developing lactic acid bacteria and lipid-reducing functional foods in the Highlands.

## 2. Materials and Methods

### 2.1. Culture and Preparation of LF-166

The target strain was isolated from traditional Tibetan yak yogurt and identified as LF-166 (CGMCC NO.25595) by 16S rRNA. It was preserved in the College of Food Science and Technology, Southwest Minzu University. LF-166 was activated twice using Man Rogosa Sharpe (MRS) broth (Hopebio, Qingdao, China), then centrifuged (1789× *g*, 10 min, 4 °C) and washed twice with saline to retrieve the bacteriophage. After resuspension of LF-166, the suspension concentration of LF-166 was adjusted to 1 × 10^8^, 1 × 10^9^, and 1 × 10^10^ CFU/mL to prepare the gavage for mice [13].

### 2.2. Animal Experiments

All animal experiments were approved by the Institutional Animal Care and Ethics Committee of Southwest Minzu University (SMU-202301145). All methods were carried out in accordance with relevant guidelines and regulations.

**Figure 1:**
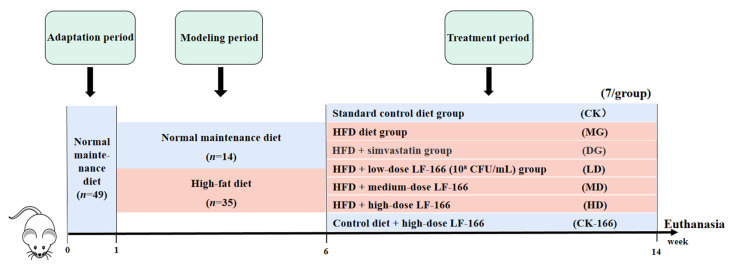
Experimental design (14 weeks).

Forty-nine SPF male Kunming mice (4-week-old, 28.3 ± 2 g) were obtained from the Chengdu Dossy Laboratory Animal Co., Ltd., Chengdu, China (SCXK (Chuan) 2020–0030). After adaptation for one week, mice were randomly divided into standard diet (*n* = 14) and high-fat (*n* = 35) diet groups (Figure 1). The standard diet group received the normal maintenance diet and the high-fat-diet group received the high-fat diet (D12492) for five weeks (a duration of 5 weeks was chosen based on our laboratory’s previous studies and related literature, suggesting that this time frame would allow for detectable differences between the group fed the basal diet and the group fed the high-fat diet [14]). Detailed compositions of the experimental diets are shown in the Supplementary Data. Blood was collected from the orbital venous plexus, and total cholesterol (TC) content was measured in the serum to evaluate hyperlipidemia. An ANOVA confirmed significant differences (*p* < 0.05) between the high-fat-diet (HFD) group and the standard diet group before treatment began (see Supplementary Data). Afterward, the mice were randomly divided into seven groups (*n* = 7): (1) standard control diet group (CK): standard diet with 1 mL/100 g saline; (2) HFD group (MG): high-fat diet with 1 mL/100 g saline; (3) HFD + simvastatin group (DG): high-fat diet with 1 mL/100 g saline and 0.36 mg/mL simvastatin; (4) HFD + low-dose LF-166 (10^8^ CFU/mL) group (LD): high-fat diet with 1 mL/100 g saline and 10^8^ CFU/mL LF-166; (5) HFD + medium-dose LF-166 (MD): high-fat diet with 1 mL/100 g saline and 10^9^ CFU/mL LF-166; (6) HFD + high-dose LF-166 (HD): high-fat diet with 1 mL/100 g saline and 10^10^ CFU/mL LF-166; and (7) control diet + high-dose LF-166 (CK-166): standard diet with 1 mL/100 g saline and 10^10^ CFU/mL LF-166. Simvastatin, as a widely used statin, has important clinical value in reducing cholesterol levels and preventing cardiovascular diseases. Therefore, including it in this research not only helps to verify its efficacy and safety under specific conditions, but also provides strong evidence and support for its use in clinical practice.

Gavage was the administration route for treatment (e.g., probiotic, simvastatin, or saline). The LF-166 and simvastatin were dissolved in saline, and the mice were gavaged at 9 A.M. daily during the experiment. Mice had ad libitum access to food and water during the feeding period. The treatment lasted eight weeks. The mice’s body weights were weighed and recorded weekly and their feed intake (see Supplementary Data) was determined until the end of the experiment. At the end of the experiment, the mice were fasted for 12 h, and blood was collected via retro-orbital puncture using a syringe with a needle under anesthesia and stored in an anticoagulation tube. After blood collection, the mice were immediately killed by cervical dislocation. The liver, epididymal and perirenal adipose, and other tissues were collected and weighed. A portion of the liver sample was fixed in 10% formaldehyde to undergo hematoxylin and eosin (H&E) staining. The remaining samples were immediately stored at −80 °C [15]. In addition, organ indices were calculated using the following equation: organ index = organ mass (mg)/mouse body mass (g) [16]. Lee’s index was determined according to the following formula [17]: Lee’s index = (body weight (g) × 1000/body length (cm))^1/3^.

### 2.3. Determination of Lipid Profile in Serum and Liver Tissues

The collected blood was centrifuged at 4 °C at 3000 rpm for 10 min. Serum was used to measure lipid profiles, including triglycerides (TG), total cholesterol (TC), high-density lipoprotein cholesterol (HDL-C), and low-density lipoprotein cholesterol (LDL-C). The liver tissue was homogenized using a glass tissue homogenizer, and then the homogenized sample was centrifuged at 4000 rpm/min for 10 min to aspirate the supernatant. The serum and tissue homogenate indicators were determined based on the kit’s instructions (Nanjing Jiancheng Bioengineering Institute, Nanjing, China).

### 2.4. Pathological Observation of Liver Tissue and Colonic Tissue

After the mouse liver and colon tissues were fixed in 10% formalin solution for 48 h, they were dehydrated, waxed, embedded, sectioned (3 μm; Leica-2016, Rotary Slicer, Hamburg, Germany), and stained with hematoxylin and eosin (H&E) for observation under a light microscope (100×) [11]. A qualitative analysis was performed to describe observed morphological differences.

### 2.5. RT-qPCR

Total RNA was extracted using TRIzol (Biosharp, Hefei, China), and a UV spectrophotometer was used to measure the total RNA concentration. The RNA was reverse-transcribed to cDNA based on the reverse transcription kit’s instructions (Tiangen, Beijing, China) and then amplified using real-time RT-qPCR. The qPCR reaction procedure was as follows: 120 s at 95 °C; 40 cycles of 15 s at 95 °C, 20 s at 55 °C, and 30 s at 72 °C; and a final cycle of 10 s at 95 °C, 60 s at 65 °C, and 1 s at 97 °C. The primer sequence of qPCR is shown in Table 1. The relative expression of the target gene was calculated with the following formula: 2^−ΔΔCt^ = 2^−[(Ct target gene − Ct housekeeping gene) − (Ct target gene − Ct housekeeping gene)]^ [18].

### 2.6. Gut Microbiota Analysis

The DNA kit was used to extract the colonic content’s microbial genome DNA. The forward primer 338F:5′-ACTCCTACGGGAGGCAGCAG-3′ and the reverse primer 806R:5′-GGACTACHVGGGTWTCTAAT-3′ were applied to amplify the V3–V4 region of the 16SrRNA gene. According to standard protocols, the library construction and sequencing were performed on an Illumina MiSeq platform by Shanghai BIOZERON Co., Ltd. (Shanghai, China).

### 2.7. Statistical Analysis

Measurement of the serum and tissue samples was conducted three times in parallel, and the mean value was then calculated. The data were processed and analyzed by Excel 2021, GraphPad Prism 9, and SPSS 20.0. The normality test determined that the data conform to a normal distribution; significant differences were analyzed by Duncan’s method in a pairwise comparison of one-way ANOVA. *p* < 0.05 corresponds to a significant difference among the groups.

## 3. Results

### 3.1. Effect of LF-166 on Growth Status and Body Weight in Mice

**Figure 2:**
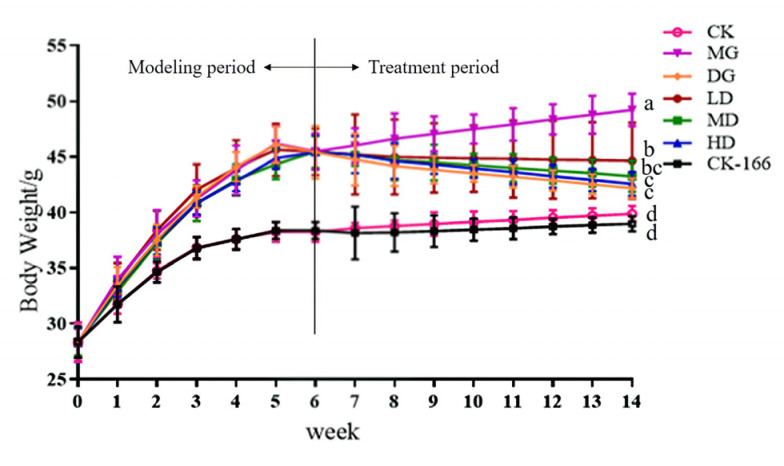
Effect of LF-166 on body weight gain in mice. Data are presented as the mean ± standard deviation (*n* = 7/group). Repeated measures ANOVA for time and treatment effects includes time as a repeated measure and treatment group as a factor. Sample data in each group have a normal distribution. Difference in variance between the two groups was significant (*p* < 0.05). ^a–d^ Mean values with different letters are significantly different (*p* < 0.05) according to Tukey’s honestly significant difference test.

The mice’s body weights showed a consistent and gradual increase during the continuous gavage (Figure 2). The average initial body weight across the groups showed no significant differences (28.3 ± 2 g). Over time, the mice’s body weights increased, with a significant rise observed in the high-fat diet (HFD) group, indicating potential fat accumulation due to the diet. After eight weeks of intervention, the body weight of the MG group continued to increase steadily, reaching the highest level among all the groups. The total body weight gained was 21.10 ± 2.36 g (see Supplementary Data). The long-term high-fat diet caused the mice to continue to gain weight, consistent with the previous results showing weight changes. During the experiment, the body weights of the mice in the CK group were significantly lower than those of the mice within the MG group. However, after the high-fat diet mice received a bacterial liquid and drug intervention, the weight gain of the LD group, MD group, HD group, and DG group mice was well controlled. The body weights of these mice were significantly lower (*p* < 0.05) than those of the MG group mice. This suggests that LF-166 administration may regulate weight gain in high-fat-diet-fed mice, showing a weight-reducing effect comparable to simvastatin.

### 3.2. Organ Indices of Mice

The experimental results (Table 2) indicated that the liver index, epididymal adipose index, and perirenal adipose index of the HFD control group were significantly elevated (*p* < 0.05) compared to those of the other groups. However, compared to the MG group, LF-166 and medication reduced the hepatic index, epididymal fat index, and perirenal fat index.

### 3.3. Effect of LF-166 on Serum and Liver Lipid Level in Mice

**Figure 3:**
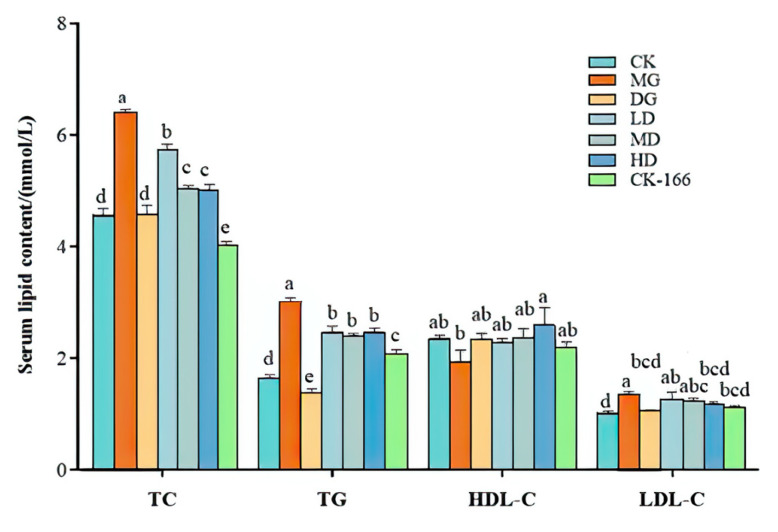
Effects of LF-166 on lipid levels in mice. Total cholesterol (TC), triglyceride (TG), high-density lipoprotein cholesterol (HDL-C), and low-density lipoprotein cholesterol (LDL-C) levels in serum of mice (*n* = 7). Data presented are the mean ± standard deviation (*n* = 7/group). Sample data in each group have a normal distribution. Difference in variance between the two groups is significant (*p* < 0.05). ^a–e^ Mean values with different letters over the same column are significantly different (*p* < 0.05) according to Tukey’s honestly significant difference test.

As shown in Figure 3, the HDL-C was significantly increased only in the high-dose LF-166 group compared to that in the MG group (*p* < 0.05). On the contrary, the HDL-C level in the serum increased significantly (*p* < 0.05). With the LF-166 solution intervention, the dyslipidemia improved to varying degrees. Compared with the MG group, the TC and TG contents in the LD group, MD group, and HD group decreased significantly (*p* < 0.05). The LDL-C in the HD group decreased significantly (*p* < 0.05). The HD group did not display significant differences between the simvastatin and normal diet groups. Simultaneously, the HDL-C content increased significantly (*p* < 0.05). In addition, the HD group’s index is closer to that of the CK and DG groups.

After eight weeks of feeding, the effect of *Lactobacillus fermentum* 166 on the liver lipid level of the mice with high-fat-diet-induced obesity is depicted in Figure 2. The TG and TC levels in the livers of the MG group were significantly higher than those of the CK group. Compared to the MG group, the liver TC and TG of the high- (HD), medium- (MD), low-dose (LD), and simvastatin (DG) groups decreased significantly, and the TC and TG reduction in the HD group was close to that in the CK group. The results showed that the ability of the tested bacteria to reduce liver blood lipids was similar to that of simvastatin; therefore, LF-166 could regulate liver lipid metabolism. However, its regulation mechanism needs to be further examined.

### 3.4. Observation and Analysis of Liver Pathology in Mice

**Figure 4:**
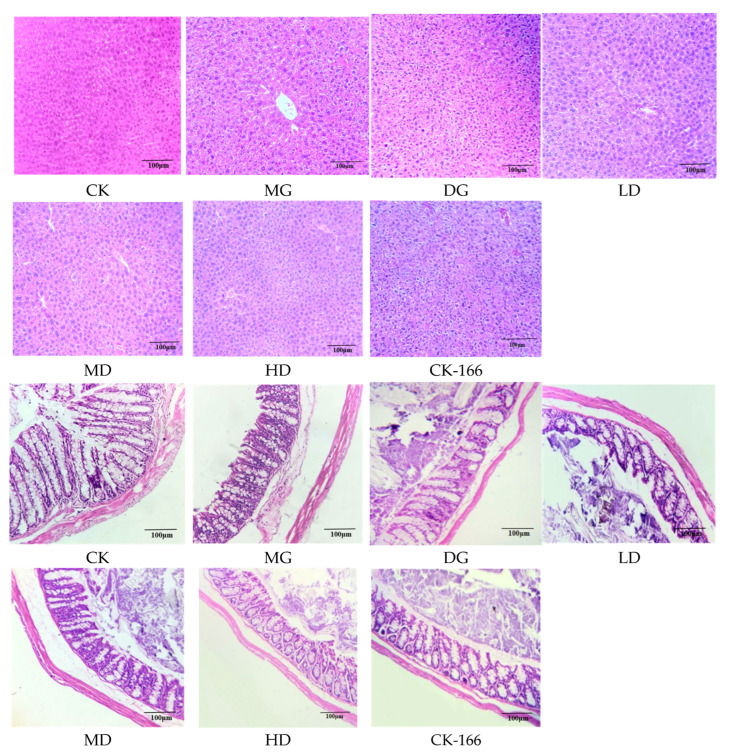
(100×) H&E pathological observation of liver and colon tissue in mice: (1) standard control diet group (CK); (2) HFD group (MG); (3) HFD + simvastatin group (DG); (4) HFD + low-dose LF-166 (10^8^ CFU/mL) group (LD); (5) HFD + medium-dose LF-166 (MD); (6) HFD + high-dose LF-166 (HD); and (7) control diet + high-dose LF-166 (CK-166).

The liver tissue structure of mice in the CK and CK-166 groups was relatively tight, complete, and transparent (Figure 4). The hepatocytes around the central portal vein were arranged in an orderly manner, the cell boundaries were clear, the cytoplasm was red, and the liver lobules were arranged regularly without steatosis, depicting a radial distribution. This indicated that the tested bacteria had no toxic side effects on the liver and would not cause damage to it. Compared with the normal CK group, the MG group’s (HFD) livers exhibited significant fat accumulation, disorganized hepatocyte cords, and large fat vacuoles, indicative of a fatty liver. When the number and size of small fat holes in the liver changed to a certain extent, a fatty liver was formed, indicating that long-term high-fat induction would cause fatty degeneration in the mice’s liver tissue. After the intervention with the drug control and bacterial solution, the fat accumulation in the liver gradually decreased, the volume of adipocytes became smaller, and the fat vacuole was significantly reduced. Fat infiltration was significantly improved, and the HD and DG group treatments were the most effective, with the improvement in steatosis in the liver being more pronounced in these mice. This suggested that LF-166 intake could reduce lipids in the liver and reduce the hepatic steatosis risk.

Colonic tissue comprises a mucous layer, submucous layer, muscular layer, and serosa. Its recess is arranged in the mucous layer, and the goblet cells are distributed in the crypts. Its integrity plays an essential role in the mucosa. As shown in Figure 4, the colonic tissue of the control group mice had a complete shape. In contrast, compared with the CK group, colonic epithelium atrophy and permeability was increased in the MG group: the colonic intima was damaged, the crypt structure was incomplete, and the villi were damaged. This indicated that a long-term high-fat diet would lead to pathological changes in the intestines of mice. In the LD, MD, and HD groups, it was observed that the bacterial solution could improve the integrity of the colon, the exfoliation of colonic epithelial cells, and the number of goblet cells, and could protect the crypt structures and intestinal villi in high-fat-diet-fed mice.

### 3.5. Effect of LF-166 on Genes Associated with Lipid Metabolism in Mice

**Figure 5:**
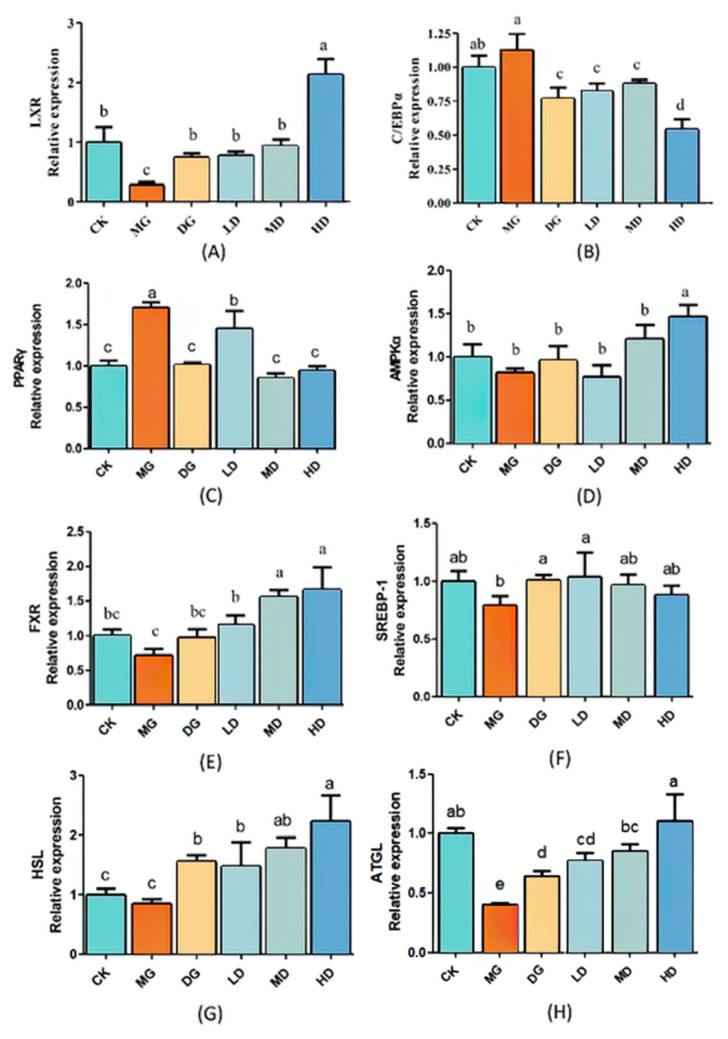
*Lxr* (**A**), *C*/*ebpα* (**B**), *Pparγ* (**C**), *Ampkα* (**D**), *Fxr* (**E**), *Srebp1* (**F**), *Hsl* (**G**), and *Atg* (**H**) mRNA expression in hepatic tissue of mice. Sample data in each group have a normal distribution, and the difference in variance between the two groups is significant (*p* < 0.05). ^a–d^ Mean values with different letters in the bar are significantly different (*p* < 0.05) according to Tukey’s honestly significant difference test.

As shown in Figure 5, the *Lxr*, *Ampkα*, and *Fxr* expression levels were down-regulated in the MG group compared to in the CK group, except for *Ampkα*, which showed no significant difference. In addition, the expression levels of the *Cebp*/*α* [19] and *Pparγ* genes were up-regulated, but Cebp/α did not show significant differences (Figure 5). In contrast, these genes revealed opposite expression trends in the control group (CK). Treatment with simvastatin and different concentrations of LF-166 decreased the expression levels of *Cebp*/*α* and *Pparγ* in the liver tissue of the hyper-lipidemic mice. There was a significant improvement in the effects of high-dose LF-166 (HD) compared to low-dose LF-166 (LD). In combination, LF-166 reduced the high-fat-diet-induced hyperlipidemia in the mice by modulating the liver mRNA expression.

**Figure 6:**
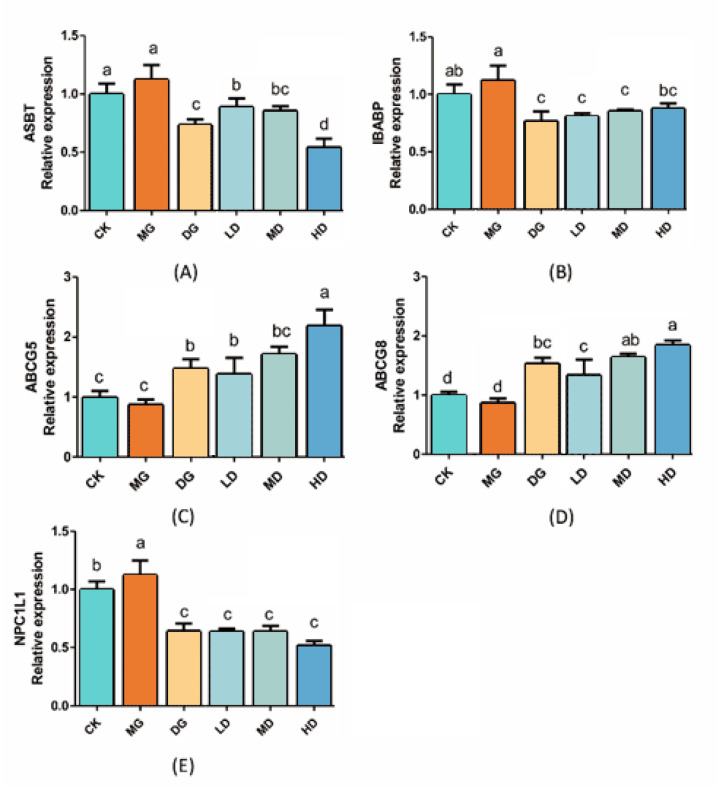
*Asbt* (**A**), *Ibabp* (**B**), *Abcg5* (**C**), *Abcg8* (**D**), and *Npc1l1* (**E**) mRNA expression in the small intestine of mice. Sample data in each group have a normal distribution, and the difference in variance between the two groups is significant (*p* < 0.05). ^a–d^ Mean values with different letters in the bar are significantly different (*p* < 0.05) according to Tukey’s honestly significant difference test.

Figure 6 indicates that the *Abcg5* and *Abcg8* gene expression levels in the HD group were significantly higher than those in the MG group (*p* < 0.05). On the contrary, the expression levels of the *Npc1l1* gene in the HD group were significantly lower (*p* < 0.05). This revealed that *Lactobacillus fermentum* 166 could up-regulate *Abcg*5 and *Abcg*8 expression and down-regulate *Npc1l1* expression in the ileum. These results showed that LF-166 regulates lipid synthesis and metabolism in hyperlipidemic mice.

All the data are expressed as means ± SD. Different letters indicate significant differences (*p* < 0.05) among the groups.

### 3.6. Effect of LF-166 on the Intestinal Microbiota in Mice

**Figure 7:**
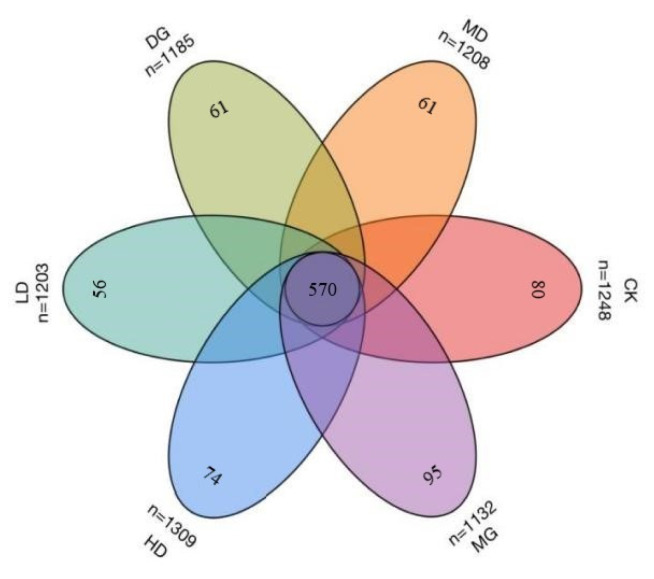
Venn diagram of shared OTUs of six samples: (1) standard control diet group (CK); (2) HFD group (MG); (3) HFD + simvastatin group (DG); (4) HFD + low-dose LF-166 (10^8^ CFU/mL) group (LD); (5) HFD + medium-dose LF-166 (MD); (6) HFD + high-dose LF-166 (HD); and (7) control diet + high-dose LF-166 (CK-166).

A Venn diagram was drawn to determine the similarity and specificity of the species distributions within each group (Figure 7). Six groups of intestinal and colon content samples produced a total of 7285 OTUs based on a sequence similarity of 97%.

The HD group had the highest number of OTUs (1309), followed by the CK (1248), MD (1208), and LD (1203) groups. The MG group had the lowest OTU count (1132). There were 570 common OTUs among the six groups. There were 95 unique OTUs in the MG group, 80 in the CK group, 61 in the DG group, 56 in the LD group, 61 in the MD and the medium LF-166 dose (MD) group, and 74 in the HD group. There was a significant increase in the number of unique OTUs after the high-dose LF-166 solution intervention in the hyperlipidemic mice, depicting a restoration of the species richness of the intestine after the probiotic intervention.

**Figure 8:**
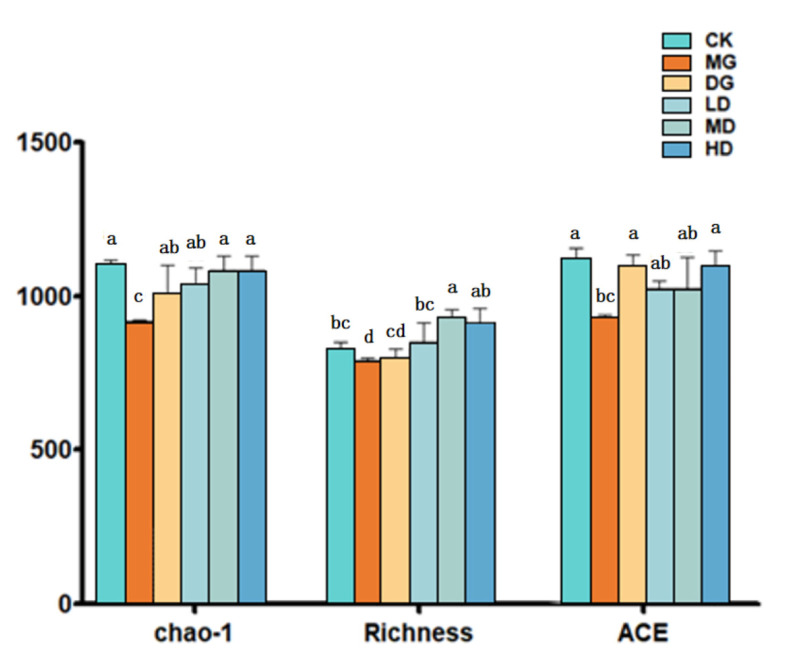
Effect of IF-166 on Alpha diversity: (1) standard control diet group (CK); (2) HFD group (MG); (3) HFD + simvastatin group (DG); (4) HFD + low-dose LF-166 (10^8^ CFU/mL) group (LD); (5) HFD + medium-dose LF-166 (MD); and (6) HFD + high-dose LF-166 (HD). Sample data in each group have a normal distribution, and the difference in variance between the two groups is significant (*p* < 0.05). ^a–d^ Mean values with different letters in the bar are significantly different (*p* < 0.05) according to Tukey’s honestly significant difference test.

A species richness and uniformity survey among the samples was conducted using the α-diversity. Compared with the CK group, the Chao1, ACE richness, and richness index (Figure 8) of the intestinal tract in the MG group were significantly reduced (*p* < 0.05), suggesting that a high-fat diet could significantly decrease the abundance and diversity of the colon contents in mice. However, the intestinal Chao, ACE, and richness indices of the mice in the LD, MD, and HD groups were higher than those in the high-fat model group, suggesting that the intestinal flora diversity was enhanced. In addition, the abundance and diversity of the intestinal microbial species were recovered.

**Figure 9:**
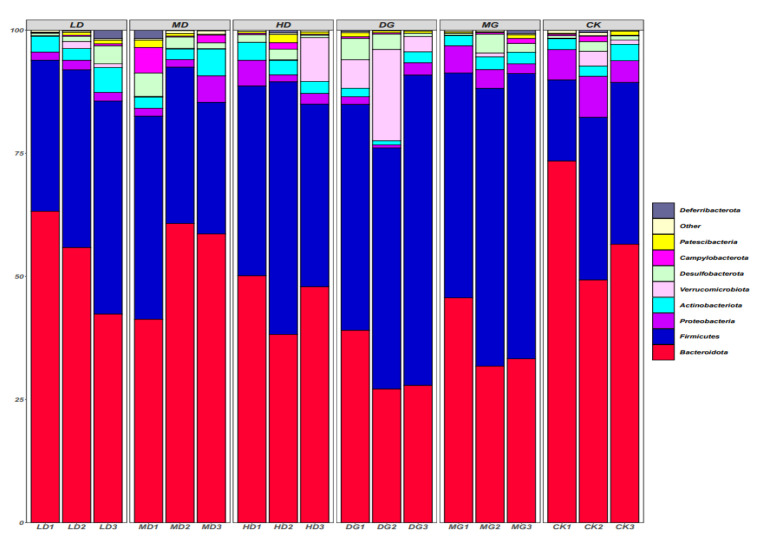
Impact of IF-166 on species distribution maps at the gate level: (1) standard control diet group (CK); (2) HFD group (MG); (3) HFD + simvastatin group (DG); (4) HFD + low-dose LF-166 (10^8^ CFU/mL) group (LD); (5) HFD + medium-dose LF-166 (MD); (6) HFD + high-dose LF-166 (HD); and (7) control diet + high-dose LF-166 (CK-166).

The results demonstrated that *Lactobacillus fermentum* 166 could improve the abundance and diversity of intestinal microflora in mice (Figure 9). It promoted the development of the intestinal microflora structure for countering the adverse effects of lipid metabolism disorders [20].

The phylum-level changes in the gut microbiota of the mice are illustrated in Figure 9. Approximately 75% of the intestinal microbiota comprises *Firmicutes* and *Bacteroidetes*. Compared to the CK group, *Firmicutes* decreased significantly, *Bacteroidetes* increased significantly, and the F/B ratio decreased in the MG group. Due to the LF-166 intervention, there were significant changes in the gut microbiota structure. This suggests that LF-166 alters the structural composition of the gut microbiota to alleviate hyperlipidemia in mice.

**Figure 10:**
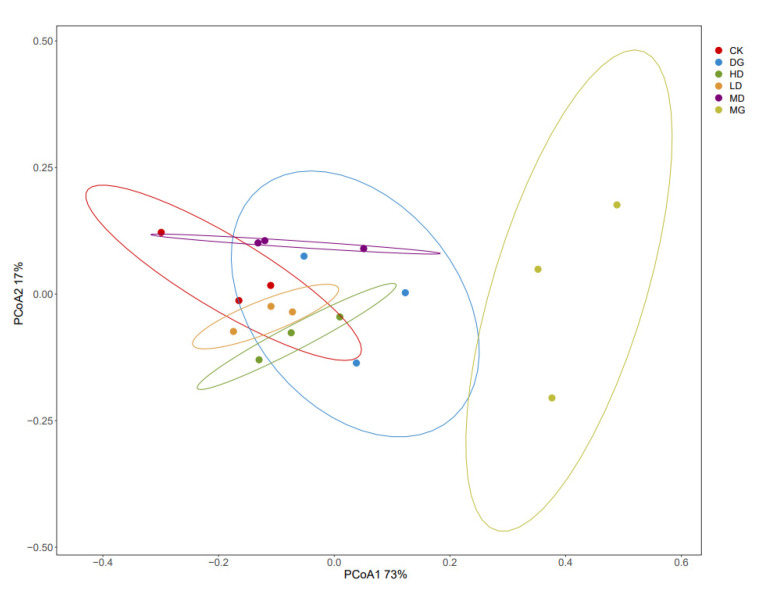
PCoA analysis of OTU level.

Figure 10 shows the PCoA, with good variation among the six sample groups. Although the sample groups were not tightly clustered, the colonic contents of the six groups developed distinct populations. The population of the MG group was wholly separated from that of the CK group, indicating that the long-term high-fat diet caused dramatic changes within the colonic intestinal flora of the mice. The LF-166 intervention decreased the distance between the samples, and the flora structure gradually returned to that of the CK group after the LF-166 intervention. Based on these results, it can be seen that LF-166 effectively reshaped the gut microbiota of the mice affected by a high-fat diet.

## 4. Discussion

High-fat diets are associated with fat accumulation, disrupted lipid metabolism, and an increased risk of cardiovascular diseases [21]. Probiotics, such as LF-166, may mitigate these effects by modulating the gut microbiota and lipid metabolism.

Like the BMI, Lee’s index reflects obesity at different levels, with a higher value indicating more obesity. In this study, the LF-166 solution inhibited an increase in Lee’s index, the liver weight index, and the fat index caused by a high-fat diet. HE staining is an effective method for observing the pathological structure of the liver and colon tissue. These results indicated that LF-166 has beneficial effects in inhibiting weight gain and reducing fat accumulation in hyperlipidemic mice. LF-166 significantly reduced (*p* < 0.05) the serum concentrations of TC, TG, and LDL-C induced by high-fat diets and also the liver TC and TG lipid levels.

Liver and ileum-based transcription factors regulate lipid anabolism and catabolism. The body maintains cholesterol homeostasis through biosynthesis [22], intestinal absorption, and bile excretion. The current paper investigated the mRNA expression of several metabolic biomarkers in the liver and ileum. *Lxrs* (Liver X Receptors) are one of the types of receptors belonging to the heterodimeric superfamily of human nuclear receptors, which are abundantly expressed in the mouse liver [23]. As one of the target genes of *Lxrs*, *Cyp7a1* promotes cholesterol absorption by the liver and enhances *Abca1* protein expression, thereby reducing cholesterol accumulation. *Ppar* can also synergistically function with *Lxrs* in HDL-C biosynthesis. In this study, the expression of *Lxr* mRNA was highest in the HD group, indicating that LF-166 may exert a lipid-lowering effect by regulating the *Ppar-Lxrα-Cyp7a1/Abca1* signaling pathway [24] that is potentially mediated by FXR activation. In this study, the up-regulation of *Fxr* mRNA [25] showed no significant effect on the *Srebp-1* mRNA expression level [26], suggesting that LF-166 did not regulate hepatic lipid metabolism via the *Fxr-Shp-Srebp-1* pathway, but through other mechanisms [27]. *C/ebpα* (CCAAT/enhancer binding proteins alpha) shows a more direct relationship with adipogenesis and works with *Pparγ* [28,29] in the terminal stage of adipocyte differentiation, and finally leads to the cells entering their fully differentiated state [30]. HSL is the only enzyme that limits the lipolysis rate. *Hsl* and *Atgl* in the body synergistically decompose triglycerides in adipose tissue. *Hsl* expression can promote fat decomposition in the body and regulate liver lipid metabolism. The RT-qPCR analysis revealed that the regulatory mechanism of *Lactobacillus fermentum* 166 on fat accumulation and cholesterol reduction could be as follows: the *Pparγ-Lxrα-Cyp7al/Abca1* signaling pathway is activated to enhance bile acid synthesis and promote cholesterol clearance; the activation of *Fxr* regulation inhibits de novo lipid synthesis; *Ampkα* activates *Pparα* [31] to promote fatty-acid β-oxidative decomposition, facilitating lipid decomposition; *Hsl* and *Atgl* synergistically decompose triglycerides in body adipose tissue in various ways. The mechanisms that affect the intestinal involvement of lipid metabolism could regulate *Asbt* and *Ibabp* transporters to inhibit bile acid reabsorption; control *Npc1l1* [32,33] and ezetimibe to inhibit cholesterol absorption; or regulate *Abcg5/g8* binding protein to promote cholesterol [34,35], such as by transferring it from the serum to the intestine to enable cholesterol efflux in a variety of ways.

The small intestine is an essential site for cholesterol metabolism, and Niemann-PickC1-like protein1 (*Npc1l1*) is expressed inside the epithelial cells of the duodenum, jejunum, and proximal ileum, mediating intestinal cholesterol and absorption of plant sterols. It reduces the plasma cholesterol levels by inhibiting intestinal cholesterol absorption by inhibiting *Npc1l1*. The body maintains cholesterol homeostasis through biosynthesis, intestinal absorption, and bile excretion. The mechanisms through which *Lactobacillus fermentum* 166 affects intestinal lipid metabolism could include regulating *Asbt* and *Ibabp* transporters to inhibit bile acid reabsorption [36], controlling N *Npc1l1* and regulating ezetimibe to inhibit cholesterol absorption [37], and regulating the adenosine triphosphate binding cassette transporter G5/G8 (*Abcg5/g8*) protein to remove serum cholesterol from the ileal lumen [38], promoting cholesterol efflux.

The results of the high-throughput sequencing analysis showed that the long-term high-fat diet reduced the diversity of the intestinal microbiota and affected the structure of the intestinal microorganisms [39]. The phylum-level investigation revealed that the proportion of Firmicutes was higher in the mice fed a long-term high-fat diet. However, the intestinal Firmicutes/Bacteroidetes values of the high-fat-diet-fed mice were reduced after the LF-166 intervention [40]. The results of the Alpha diversity analysis revealed that the high-fat diet affected the diversity of the intestinal microflora in the mice, and the diversity was improved after the Lactobacillus spp. intervention. Based on the results of the Beta diversity analysis, the intestinal flora structure of the CK and MG groups was significantly different due to differences in the groups’ dietary structure. Compared to the MG group, the differences between the CK group, LD group, MD group, and HD group were narrowed, suggesting that the LF-166 intervention could change the intestinal flora structure, thus alleviating the lipid metabolism disorder in hyperlipidemic mice.

## 5. Conclusions

Research shows that LF-166 can reduce liver and small intestine damage related to hyperlipidemia and improve abnormal lipid metabolism in serum and liver tissues. The potential mechanism of LF-166 was associated with the up-regulation of LXR, AMPKα, FXR, HSL, ATGL, and SREBP-1 mRNA expression, and with the down-regulation of CEBP/α and PPARγ mRNA expression in the liver tissue. LF-166 could up-regulate the expression of ABCG5 and ABCG8 in the ileum and down-regulate the expression of NPC1L1 in the ileum. The high-fat diet caused disorders in the mice’s gut microbiota. The LF-166 intervention restored the intestine’s species richness in the hyperlipidemic mice. LF-166 has been shown to have a relieving effect on hyperlipidemia and its mechanism. This means that LF-166 has the potential for further applications as a probiotic or a food. Mice share certain physiological similarities with humans, and mouse models offer a range of customizable genotypes and phenotypes that are far superior to any other model organism. As such, they play a very important role in the emerging field of gut microbiota research. But models always differ to some degree from the system that they model [41]. Future studies should investigate the molecular mechanisms underlying LF-166’s therapeutic effects and explore its potential for clinical applications.

## Figures and Tables

**Table 1:** Sequences of primers for qPCR.

Gene Name	Sequence (5′-3′)	Temperature (°C)
*Asbt*	F:5′-GTAGGGGATCACAATCGTTCCT-3′	54.7
	R:5′-ATAGCTTGGTCGTGGAGGTCACT-3′	56.9
*Ibabp*	F:5′-CAGACTTCCCCAACTATCACCAG-3′	56.9
	R:5′-TCAAGCCACCCTCTTGCTTAC-3′	54.2
*Npc1l1*	F:5′-CCCCAAACTCCCTCATAAGCA-3′	54.2
	R:5′-TATCCCCCAACAGCAAGGAAG-3′	54.2
*Abcg5*	F:5′-TTGGCCCCTCACTTAATTGGA-3′	52.2
	R:5′-GGACCATACCAAGCAGCACAAG-3′	56.5
*Abcg8*	F:5′-ACTGCCATGGACCTGAACTCA-3′	54.2
	R:5′-GCTGATGCCAATGACGATGA-3′	51.6
*Atgl*	F:5′-GGATGGCGGCATTTCAGACA-3′	62.9
	R:5′-CAAAGGGTTGGGTTGGTTCAG-3′	61.3
*Hsl*	F:5′-CCAGCCTGAGGGCTTACTG-3′	61.7
	R:5′-CTCCATTGACTGTGACATCTCG-3′	60.4
*Srebp1*	F:5′-GATGTGCGAACTGGACACAG-3′	61.0
	R:5′-CATAGGGGGCGTCAAACAG-3′	60.2
*Fxr*	F:5′-GCTAAGGAAGTGCAGAGAGATGG-3′	56.9
	R:5′-ATAGCTTGGTCGTGGAGGTCACT-3′	56.9
*Ampkα*	F:5′-ACCTGAGAACGTCCTGCTTG-3′	53.7
	R:5′-GGCCTGCGTACAATCTTCCT-3′	53.7
*Pparγ*	F:5′-GGAGCCTAAGTTTGAGTTTGCTGTG-3′	57.5
	R:5′-TGCAGCAGGTTGTCTTGGATG-3′	54.2
*C*/*ebpα*	F:5′-TGCTGGAGTTGACCAGTGACAA-3′	54.7
	R:5′-AAACCATCCTCTGGGTCTCC-3′	53.7
*Lxr*	F:5′-CATCAAGGGAGCACGCTACATT-3′	54.7
	R:5′-GCATTTGCGAAGGCGACAC-3′	53.1
*β-actin*	F:5′-ACGGTCAGGTCATCACTATCG-3′	54.2
	R:5′-GGCATAGAGGTCTTTACGGATG-3′	54.7

**Table 2:** Organ indices of mice in each group (n = 7).

Group	Liver Index	Epididymal Adipose Index	Perirenal Adipose Index
CK	40.17 *±* 0.22 ^d^	15.45 *±* 0.67 ^c^	5.59 *±* 1.00 ^c^
MG	50.65 *±* 0.61 ^a^	25.43 *±* 1.38 ^a^	8.97 *±* 0.77 ^a^
DG	42.09 *±* 0.84 ^cd^	15.46 *±* 1.04 ^c^	6.98 *±* 0.56 ^bc^
LD	44.65 *±* 1.39 ^b^	19.21 *±* 1.00 ^b^	7.61 *±* 1.17 ^ab^
MD	42.47 *±* 1.35 ^c^	16.44 *±* 1.81 ^c^	6.53 *±* 0.68 ^bc^
HD	41.10 *±* 0.70 ^cd^	14.51 *±* 0.35 ^c^	5.32 *±* 0.46 ^c^
CK-166	40.31 *±* 0.34 ^d^	15.44 *±* 0.67 ^c^	5.65 *±* 0.99 ^c^

Values presented are the mean ± standard deviation (*n* = 7/group). Sample data in each group have a normal distribution. Difference in variance between the two groups was significant (*p* < 0.05). ^a–d^ Mean values with different letters over the same column are significantly different (*p* < 0.05) according to Tukey’s honestly significant difference test.

## Data Availability

The original contributions presented in this study are included in the article, and further inquiries can be directed to the corresponding authors.

## Supplementary Materials

The following supporting information can be downloaded at: https://www.mdpi.com/article/10.3390/foods14050867/s1, Figure S1: Serum TC of mice after 5 weeks; Table S1: Nutrient Composition Table of Mouse Diet; Table S2: The food intake; Table S3. the weight changes over eight weeks.

## Abbreviations

The following abbreviations are used in this manuscript:

TG	triglycerides
TC	total cholesterol
HDL-C	high-density lipoprotein cholesterol
LDL-C	low-density lipoprotein cholesterol
HFD	high-fat diet
*Lxr*	liver X receptors
*C/ebpα*	CCAAT Enhancer Binding Protein Alpha
*Pparγ*	Peroxisome Proliferator Activated Receptor Gamma
*Ampk α*	AMP-Activated Protein Kinase Alpha
*Fxr*	Farnesoid X Receptor
*Srebp-1*	Sterol Regulatory Element Binding Protein 1
*Hsl*	Hormone-sensitive lipase
*Atgl*	Adipose Triglyceride Lipase
*Asbt*	Apical Sodium-dependent Bile acid Transporter
*Ibabp*	ileum Bile Acid Binding Protein
*Abcg5*	ATP Binding Cassette subfamily G member 5
*Abcg8*	ATP Binding Cassette subfamily G member 8
*Npc1l1*	NPC1 Like intracellular cholesterol transporter 1

## References

[1] Lusis A.J. (2000). Atherosclerosis. Nature.

[2] Leong D.P., Joseph P.G., McKee M., Anand S.S., Teo K.K., Schwalm J.D., Yusuf S. (2017). Reducing the Global Burden of Cardiovascular Disease, Part 2: Prevention and Treatment of Cardiovascular Disease. Circ. Res..

[3] Duong M., Islam S., Rangarajan S., Leong D., Kurmi O., Teo K., Killian K., Dagenais G., Lear S., Wielgosz A. (2019). Mortality and cardiovascular and respiratory morbidity in individuals with impaired FEV1 (PURE): An international, community-based cohort study. Lancet Glob. Health.

[4] Gluchowski N.L., Becuwe M., Walther T.C., Farese R.V. (2017). Lipid droplets and liver disease: From basic biology to clinical implications. Nat. Rev. Gastroenterol. Hepatol..

[5] Mkadem W., Belguith K., Oussaief O., ElHatmi H., Indio V., Savini F., De Cesare A., Boudhrioua N. (2023). Systematic approach to select lactic acid bacteria from spontaneously fermented milk able to fight Listeria monocytogenes and Staphylococcus aureus. Food Biosci..

[6] Sittipo P., Lobionda S., Lee Y.K., Maynard C.L. (2018). Intestinal microbiota and the immune system in metabolic diseases. J. Microbiol..

[7] Miao G.L., Guo J.B., Zhang W.X., Lai P.P., Xu Y.T., Chen J.X., Zhang L.X., Zhou Z.H., Han Y.F., Chen G.L. (2024). Remodeling Intestinal Microbiota Alleviates Severe Combined Hyperlipidemia-Induced Nonalcoholic Steatohepatitis and Atherosclerosis in LDLR-/- Hamsters. Research.

[8] Fan X.Y., Zhang Q., Guo W.L., Wu Q., Hu J.P., Cheng W.J., Lue X.C., Rao P.F., Ni L., Chen Y.T. (2023). The protective effects of *Levilactobacillus brevis* FZU0713 on lipid metabolism and intestinal microbiota in hyperlipidemic rats. Food Sci. Hum. Wellness.

[9] Li H., Ma Y., Li Q., Wang J., Cheng J., Xue J., Shi J. (2011). The chemical composition and nitrogen distribution of Chinese yak (Maiwa) milk. Int. J. Mol. Sci..

[10] Feng F., Yang G., Ma X., Zhang J., Huang C., Ma X., La Y., Yan P., Zhandui P., Liang C. (2024). Polymorphisms within the PRKG1 Gene of Gannan Yaks and Their Association with Milk Quality Characteristics. Foods.

[11] Zhu K., Tan F., Mu J., Yi R., Zhou X., Zhao X. (2019). Anti-Obesity Effects of Lactobacillus fermentum CQPC05 Isolated from Sichuan Pickle in High-Fat Diet-Induced Obese Mice through PPAR-α Signaling Pathway. Microorganisms.

[12] Qian Y., Song J.-L., Yi R., Li G., Sun P., Zhao X., Huo G. (2018). Preventive effects of Lactobacillus plantarum YS4 on constipation induced by activated carbon in mice. Appl. Sci..

[13] Liu Z., Zhao J., Sun R., Wang M., Wang K., Li Y., Shang H., Hou J., Jiang Z. (2022). Lactobacillus plantarum 23-1 improves intestinal inflammation and barrier function through the TLR4/NF-κB signaling pathway in obese mice. Food Funct..

[14] Lu Y., Sun W., Zhang Z., Yu J., Zhang J., Guo Q. (2025). Lactiplantibacillus plantarum A5 alleviates high-fat diet-induced hyperlipidemia via regulating gut microbiota to promote short-chain fatty acids production. Food Biosci..

[15] Qu L.L., Yu B., Li Z., Jiang W.X., Jiang J.D., Kong W.J. (2016). Gastrodin Ameliorates Oxidative Stress and Proinflammatory Response in Nonalcoholic Fatty Liver Disease through the AMPK/Nrf2 Pathway. Phytother. Res..

[16] Zhong Y., Zhang X., Hu X., Li Y. (2018). Effects of Repeated Lipopolysaccharide Treatment on Growth Performance, Immune Organ Index, and Blood Parameters of Sprague-Dawley Rats. J. Vet. Res..

[17] Teng Y., Wang Y., Tian Y., Chen Y.-y., Guan W.-y., Piao C.-h., Wang Y.-h. (2020). Lactobacillus plantarum LP104 ameliorates hyperlipidemia induced by AMPK pathways in C57BL/6N mice fed high-fat diet. J. Funct. Foods.

[18] Chen Q.-Q., Liu K., Shi N., Ma G., Wang P., Xie H.-M., Jin S.-J., Wei T.-T., Yu X.-Y., Wang Y. (2023). Neuraminidase 1 promotes renal fibrosis development in male mice. Nat. Commun..

[19] Quiroz-Figueroa K., Vitali C., Conlon D.M., Millar J.S., Tobias J.W., Bauer R.C., Hand N.J., Rader D.J. (2021). TRIB1 regulates LDL metabolism through CEBPα-mediated effects on the LDL receptor in hepatocytes. J. Clin. Investig..

[20] Hildebrandt M.A., Hoffmann C., Sherrill-Mix S.A., Keilbaugh S.A., Hamady M., Chen Y.Y., Knight R., Ahima R.S., Bushman F., Wu G.D. (2009). High-fat diet determines the composition of the murine gut microbiome independently of obesity. Gastroenterology.

[21] Geng J., Zhang X., Guo Y., Wen H., Guo D., Liang Q., Pu S., Wang Y., Liu M., Li Z. (2025). Moderate-intensity interval exercise exacerbates cardiac lipotoxicity in high-fat, high-calories diet-fed mice. Nat. Commun..

[22] Liu H., Pathak P., Boehme S., Chiang J.L. (2016). Cholesterol 7α-hydroxylase protects the liver from inflammation and fibrosis by maintaining cholesterol homeostasis. J. Lipid Res..

[23] Wang B., Tontonoz P. (2018). Liver X receptors in lipid signalling and membrane homeostasis. Nat. Rev. Endocrinol..

[24] Chen Z., Shao W., Li Y., Zhang X., Geng Y., Ma X., Tao B., Ma Y., Yi C., Zhang B. (2024). Inhibition of PCSK9 prevents and alleviates cholesterol gallstones through PPARα-mediated CYP7A1 activation. Metabolism.

[25] Jiang B., Yuan G., Wu J., Wu Q., Li L., Jiang P. (2022). Prevotella copri ameliorates cholestasis and liver fibrosis in primary sclerosing cholangitis by enhancing the FXR signalling pathway. Biochim. Biophys. Acta Mol. Basis Dis..

[26] Jin M., Shen Y., Pan T., Zhu T., Li X., Xu F., Betancor M.B., Jiao L., Tocher D.R., Zhou Q. (2021). Dietary Betaine Mitigates Hepatic Steatosis and Inflammation Induced by a High-Fat-Diet by Modulating the Sirt1/Srebp-1/Pparɑ Pathway in Juvenile Black Seabream (*Acanthopagrus schlegelii*). Front. Immunol..

[27] Horton J.D., Goldstein J.L., Brown M.S. (2002). SREBPs: Activators of the complete program of cholesterol and fatty acid synthesis in the liver. J. Clin. Investig..

[28] Janani C., Ranjitha Kumari B.D. (2015). PPAR gamma gene—A review. Diabetes Metab. Syndr..

[29] Choi J.S., Kim J.H., Ali M.Y., Min B.S., Kim G.D., Jung H.A. (2014). Coptis chinensis alkaloids exert anti-adipogenic activity on 3T3-L1 adipocytes by downregulating C/EBP-α and PPAR-γ. Fitoterapia.

[30] Braissant O., Foufelle F., Scotto C., Dauça M., Wahli W. (1996). Differential expression of peroxisome proliferator-activated receptors (PPARs): Tissue distribution of PPAR-alpha, -beta, and -gamma in the adult rat. Endocrinology.

[31] Janssen A.W., Betzel B., Stoopen G., Berends F.J., Janssen I.M., Peijnenburg A.A., Kersten S. (2015). The impact of PPARα activation on whole genome gene expression in human precision cut liver slices. BMC Genom..

[32] Altmann S.W., Davis H.R., Zhu L.J., Yao X., Hoos L.M., Tetzloff G., Iyer S.P., Maguire M., Golovko A., Zeng M. (2004). Niemann-Pick C1 Like 1 protein is critical for intestinal cholesterol absorption. Science.

[33] Jia L., Betters J.L., Yu L. (2011). Niemann-pick C1-like 1 (NPC1L1) protein in intestinal and hepatic cholesterol transport. Annu. Rev. Physiol..

[34] Yang C., Yu L., Li W., Xu F., Cohen J.C., Hobbs H.H. (2004). Disruption of cholesterol homeostasis by plant sterols. J. Clin. Investig..

[35] Kawase A., Hata S., Takagi M., Iwaki M. (2015). Pravastatin Modulate Niemann-Pick C1-Like 1 and ATP-Binding Cassette G5 and G8 to Influence Intestinal Cholesterol Absorption. J. Pharm. Pharm. Sci..

[36] Out C., Patankar J.V., Doktorova M., Boesjes M., Bos T., de Boer S., Havinga R., Wolters H., Boverhof R., van Dijk T.H. (2015). Gut microbiota inhibit Asbt-dependent intestinal bile acid reabsorption via Gata4. J. Hepatol..

[37] Hoang M.H., Houng S.J., Jun H.J., Lee J.H., Choi J.W., Kim S.H., Kim Y.R., Lee S.J. (2011). Barley intake induces bile acid excretion by reduced expression of intestinal ASBT and NPC1L1 in C57BL/6J mice. J. Agric. Food Chem..

[38] Yu L., Li-Hawkins J., Hammer R.E., Berge K.E., Horton J.D., Cohen J.C., Hobbs H.H. (2002). Overexpression of ABCG5 and ABCG8 promotes biliary cholesterol secretion and reduces fractional absorption of dietary cholesterol. J. Clin. Investig..

[39] Cresci G.A., Bawden E. (2015). Gut Microbiome: What We Do and Don’t Know. Nutr. Clin. Pract..

[40] Murphy E.F., Cotter P.D., Healy S., Marques T.M., O’Sullivan O., Fouhy F., Clarke S.F., O’Toole P.W., Quigley E.M., Stanton C. (2010). Composition and energy harvesting capacity of the gut microbiota: Relationship to diet, obesity and time in mouse models. Gut.

[41] Nguyen T.L., Vieira-Silva S., Liston A., Raes J. (2015). How informative is the mouse for human gut microbiota research?. Dis. Model. Mech..

