# Supplementary material for: Lactobacillus fermentum 166, Derived from Yak Yogurt from Tibetan Areas of Sichuan, Improves High-Fat-Diet-Induced Hyperlipidemia by Modulating Gut Microbiota and Liver- and Gut-Related Pathways
Source: Foods. 2025 Mar 3;14(5):867. doi: 10.3390/foods14050867 (PMC11898959; doi:10.3390/foods14050867)
Supplement: Supplementary file 1 [file foods-14-00867-s001.zip › foods-3447634-supplementary.pdf]

Table S1. Nutrient Composition Table of Mouse Diet

Diet Ingredients	the normal maintenance diet (gm%)	high-fat diet (D12492) (gm%)
Protein	18.50	26.00
Fat	5.20	35.00
Carbohydrate	53.00	26.00

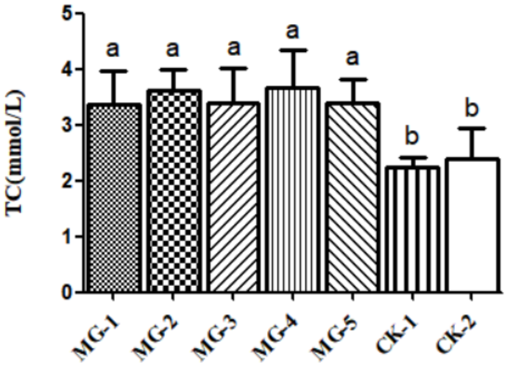

Figure S1. Serum TC of mice after 5 weeks

Table S2. The food intake

Constituencies	1st Week	2st Week	3st Week	4st Week	5st Week	6st Week	7st Week	8st Week
CK	4.50±0.15[a]	4.57±0.16[a]	4.65±0.17[a]	4.72±0.18[a]	4.80±0.19[a]	4.89±0.20[a]	4.96±0.20[a]	5.02±0.20[a]
MG	4.35±0.18[a]	4.42±0.19[a]	4.50±0.20[a]	4.58±0.21[a]	4.65±0.22[a]	4.73±0.22[a]	4.80±0.22[a]	4.85±0.22[a]
DG	4.40±0.10[a]	4.47±0.11[a]	4.55±0.12[a]	4.62±0.12[a]	4.70±0.12[a]	4.78±0.12[a]	4.85±0.12[a]	4.90±0.12[a]
LD	4.45±0.20[a]	4.52±0.21[a]	4.60±0.22[a]	4.67±0.23[a]	4.75±0.25[a]	4.83±0.26[a]	4.90±0.27[a]	4.95±0.28[a]
MD	4.55±0.22[a]	4.62±0.23[a]	4.70±0.24[a]	4.78±0.24[a]	4.85±0.25[a]	4.93±0.25[a]	5.00±0.25[a]	5.06±0.25[a]
HD	4.38±0.23[a]	4.45±0.24[a]	4.53±0.25[a]	4.60±0.26[a]	4.68±0.27[a]	4.76±0.27[a]	4.83±0.27[a]	4.88±0.27[a]
CK-166	4.48±0.17[a]	4.55±0.18[a]	4.63±0.19[a]	4.70±0.20[a]	4.78±0.21[a]	4.86±0.21[a]	4.93±0.21[a]	4.98±0.21[a]

Values presented are the mean ± standard deviation (N =7/group). Sample data in each group come from a normal distribution, and the difference in variance between the two groups was significant (p < 0.05).[a] Mean values over the same column are not significantly different (p < 0.05) according to Tukey's honestly significant difference.

Table S3. the weight changes over eight weeks

t（week）	LD	MD	HD	MG	DG	CK	CK-166
0	45.44±1.92[a]	45.45±1.29[a]	45.42±1.42[a]	45.48±1.47[a]	45.40±2.15[a]	38.25±0.82[b]	38.37±0.70[b]
1	45.22±3.29[a]	45.17±0.89[a]	45.20±1.52[a]	46.02±1.43[a]	44.77±2.12[a]	38.58±0.46[b]	38.15±2.18[b]
2	44.98±3.08[a]	44.73±1.66[a]	44.63±1.47[a]	46.63±2.09[a]	44.20±1.65[a]	38.77±0.47[b]	38.20±1.59[b]
3	44.92±2.85[b]	44.50±1.47[b]	44.33±0.78[b]	47.07±1.45[a]	43.85±1.13[b]	38.97±0.97[c]	38.32±1.31[c]
4	44.87±2.73[b]	44.27±0.91[b]	43.97±0.77[b]	47.50±1.19[a]	43.53±0.69[a]	39.15±0.85[c]	38.45±0.93[c]
5	44.82±3.18[b]	44.00±1.13[b]	43.65±1.01[b]	47.93±1.34[b]	43.22±0.89[b]	39.33±0.73[c]	38.57±0.89[c]
6	44.75±3.20[b]	43.77±1.05[b]	43.27±0.94[b]	48.38±1.26[a]	42.90±0.84[b]	39.53±0.60[c]	38.73±0.63[c]
7	44.70±3.13[a]	43.55±1.09[b]	42.93±0.92[bc]	48.80±1.57[bc]	42.53±0.85[c]	39.70±0.62[d]	38.87±0.63[d]
8	44.65±3.13[b]	43.27±1.28[bc]	42.58±1.00[c]	49.23±1.33[a]	42.20±0.83[c]	39.88±0.65[d]	38.98±0.63[d]

Values presented are the mean ± standard deviation (N = 7/group). Sample data in each group come from a normal distribution, and the difference in variance between the two groups was significant ($p < 0.05$). [a] Mean values over the same column are not significantly different ($p < 0.05$) according to Tukey's honestly significant difference.
